# The Making of a Monster: Postnatal Ontogenetic Changes in Craniomandibular Shape in the Great Sabercat *Smilodon*

**DOI:** 10.1371/journal.pone.0029699

**Published:** 2012-01-03

**Authors:** Per Christiansen

**Affiliations:** Department of Biotechnology, Chemistry, and Environmental Engineering, University of Aalborg, Aalborg, Denmark; University College London, United Kingdom

## Abstract

Derived sabercats had craniomandibular morphologies that in many respects were highly different from those of extant felids, and this has often been interpreted functionally as adaptations for predation at extreme gape angles with hypertrophied upper canines. It is unknown how much of this was a result of intraspecific postnatal ontogeny, since juveniles of sabercats are rare and no quantitative study has been made of craniomandibular ontogeny. Postnatal ontogenetic craniomandibular shape changes in two morphologically derived sabercats, *Smilodon fatalis* and *S. populator*, were analysed using geometric morphometrics and compared to three species of extant pantherines, the jaguar, tiger, and Sunda clouded leopard. Ontogenetic shape changes in *Smilodon* usually involved the same areas of the cranium and mandible as in extant pantherines, and large-scale modularization was similar, suggesting that such may have been the case for all felids, since it followed the same trends previously observed in other mammals. However, in other respects *Smilodon* differed from extant pantherines. Their crania underwent much greater and more localised ontogenetic shape changes than did the mandibles, whereas crania and mandibles of extant pantherines underwent smaller, fewer and less localised shape changes. Ontogenetic shape changes in the two species of *Smilodon* are largely similar, but differences are also present, notably those which may be tied to the presence of larger upper canines in *S. populator*. Several of the specialized cranial characters differentiating adult *Smilodon* from extant felids in a functional context, which are usually regarded as evolutionary adaptations for achieving high gape angles, are ontogenetic, and in several instances ontogeny appears to recapitulate phylogeny to some extent. No such ontogenetic evolutionary adaptive changes were found in the extant pantherines. Evolution in morphologically derived sabercats involved greater cranial ontogenetic changes than among extant felids, resulting in greatly modified adult craniomandibular morphologies.

## Introduction

Felids are some of the most anatomically specialized of all mammals for vertebrate predation, and the extinct sabertoothed felids (Felidae: Machairodontinae) included some of the most craniodentally specialized of all mammalian carnivores. Among the most morphologically derived were *Smilodon* spp., in particular the two great Pleistocene species *Smilodon fatalis* and *S. populator* from North and north-western South America, and eastern South America, respectively [1], [2]. Recently, *S. populator* has also been documented from Venezuela [3] and as far south as Chilean Patagonia [4]. Some [5], [6] have argued that they represent a single species, to which the name *S. populator* [7] would then apply, but this view has not gained ground, and there are several character differences between them pertaining, for instance, to cranial form and canine size and shape [1], [2]. A recent phylogenetic study found a further difference in that the P^4^ protocone is more reduced in *S. populator* [8].

*Smilodon* were the quintessential “sabertoothed tigers”, and craniodentally, they differed markedly from all extant felids, having, among others, greatly elongate and lateromedially flattened upper canines and greatly reduced (incisiform) lower canines; a tall, compact cranial shape; an enormously developed mastoid process and a greatly reduced paroccipital process, implying great enhancement of the cranial flexor musculature; shorter and more massive zygomatic arches; prognatheous incisors; lowered glenoid joint; and a mandible with a rectangular and straight horizontal ramus, verticalized mandibular symphysis, greatly reduced coronoid process, laterally rotated lower carnassials, and a deflected retroarticular process [2], [9], [10]–[17]. Their bite forces relative to body size were lower than among extant large felids [18]–[21], and their mandibular morphology indicated significant differences in predatory behaviour from extant felids [10], [21], [22]. The tall skull shape was likely an adaptation for re-orientation of the major mandibular adductor muscles to facilitate more efficient biting even at high gape angles and to partially compensate for the reduction in mandibular adductor muscle size [9], [10], [19]–[21]. The prognatheous incisors are thought to have facilitated carcass dismembering with very large upper canines, and perhaps as an auxiliary anchor point during predatory biting [2], [10], [11].

Extant adult felids are hyper-carnivorous and, as such, undergo a dramatic change in diet from suckling to a mechanically demanding ecology as vertebrate predators, necessitating high bite forces [23]. This transition in behaviour and functional morphology could be accompanied by large changes in craniomandibular morphology, but skull ontogeny in felids has received relatively little attention, and most studies have focused on dental eruption and subsequent wear, and its implication for age determination [24]–[31]. Other studies of postnatal morphology and size changes in lynxes (*Lynx* spp.) [32], [33]; margay (*Leopardus wiedii*) [34]; caracal (*Caracal caracal*) [35]; jaguar (*Panthera onca*) [36]; tiger (*Panthera tigris*) [37]; and lion (*Panthera leo*) [38] have been primarily descriptive. To date, the only species in which craniomandibular ontogeny has been specifically studied is the puma (*Puma concolor*) using both qualitative [39] and quantitative [40] approaches. Another study involved adult size and morphology changes [41], since growth in felids proceeds for years beyond sexual maturity, as in many other mammal species [42]–[44]. A recent study involved allometric cranial proportions in extant lions to address the nature of the Ice Age pantherine *Panthera atrox*, but did not address ontogeny as such [45].

Since some sabercats were even more craniomandibularly and dentally specialized than extant felids, studies of craniomandibular ontogeny could potentially shed light onto how sabercat adults came to be so morphologically different from modern felids. Unfortunately, postnatal ontogenetic changes in sabercat craniomandibular morphologies are even more poorly known than those of extant felids, and juveniles are known for only a few species, for instance *Homotherium serum* [46], [47] and *Smilodon fatalis* [48]; additionally, previous authors have focused on descriptive comparisons between juveniles and adults. In the voluminous collections of the Late Pleistocene fauna from the La Brea tar seeps, juvenile *Smilodon fatalis* from several dental ages are known [48], and juveniles appear not to have been rare, since Miller [49], in a study of 918 skulls, estimated that 16.6% represented juveniles; 23.2% were young adults; 17.2% were adults; and 8.5% were old individuals; 34.5% were indeterminable, but were mostly adult specimens who could not be classified as young adults, adults, or old. Most juvenile skulls are incomplete, however, owing to poor ossification and the movement of the tar. Other than Merriam & Stock [48] and Tejada-Flores & Shaw [50], the *S. fatalis* juveniles have received relatively little literature exposure.

**Figure 1:**
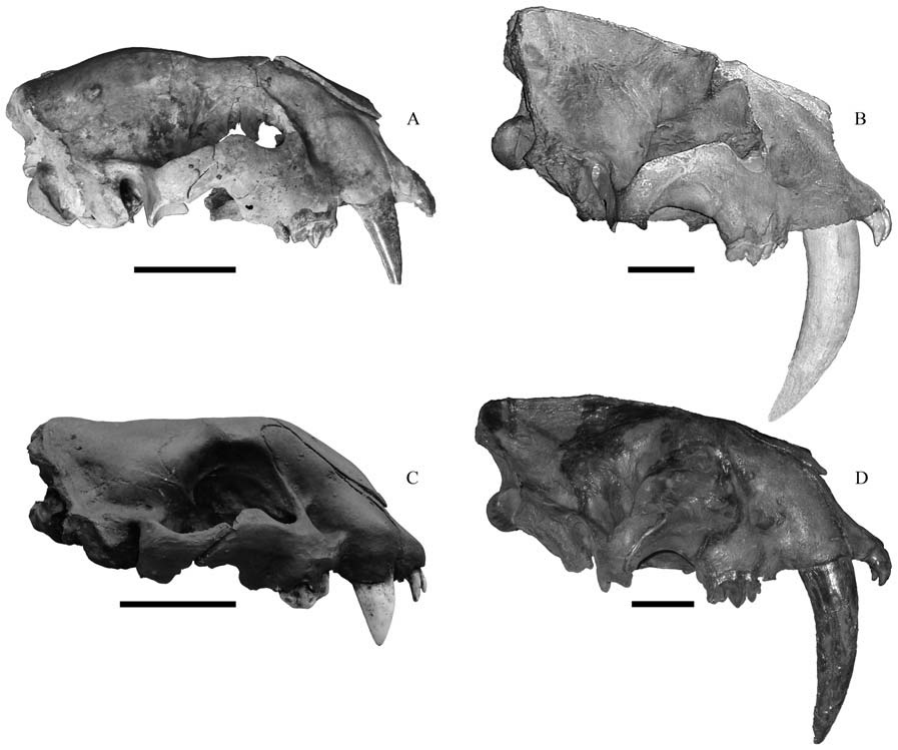
A comparison of crania in juvenile and adult *Smilodon* spp. scaled to the same condylobasal length. A, juvenile *S. populator* (NRM); B, adult *S. populator* (BM, cast); C, juvenile *S. fatalis* (PC coll.; from Dinocasts); D, adult *S. fatalis* (LACMHC2001-173). Scale bars equal 5 cm.

**Figure 2:**
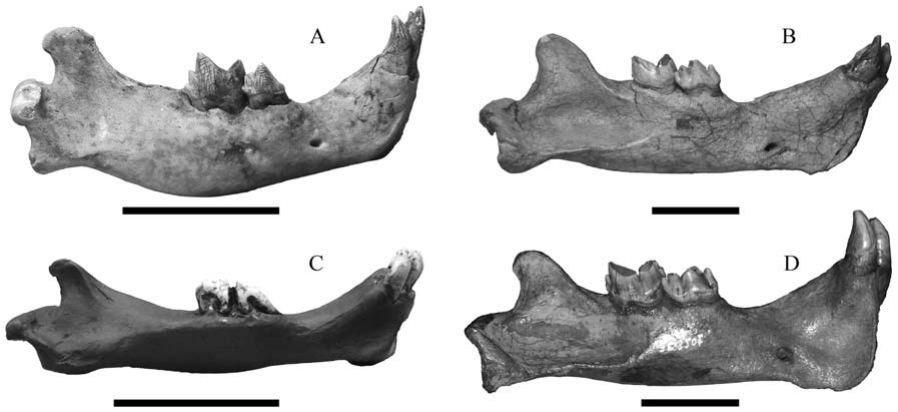
A comparison of mandibles in juvenile and adult *Smilodon* spp. scaled to the same overall length. A, juvenile *S. populator* (NRM); B, adult *S. populator* (CN52); C, juvenile *S. fatalis* (PC coll.; from Dinocasts); D, adult *S. fatalis* (LACMHC3E-350). The juvenile *S. fatalis* mandible is an example of a gracile mandible type whereas the adult mandible is of a more robust type, but variation in juveniles and adults in the La Brea material is substantial. Scale bars equal 5 cm.

*Smilodon populator* was even larger [51] and in some respects more specialized than *S. fatalis*, for instance in having an even larger C^1^ [1], [2], but only fragmentary and incomplete juvenile skulls have been reported of this species [52]. However, an exquisitely preserved juvenile specimen is present at the Naturhistoriska riksmuseet in Stockholm, (Fig. 1,2; Fig. S1), and other than a meticulous fitting together of a few skull bones around the nasals, owing to their not being co-ossified yet, the specimen is in excellent and near undistorted condition. The deciduous dentition is fully erupted and there are wear facets on the carnassials (dP^3^; dP_4_), in particular the dP_4_. The permanent dentition has not yet erupted, but the permanent carnassials (P^4^; M_1_) can be seen inside their alveoli. The left dI^3^ has fallen out indicating that the permanent incisors may soon commence replacing the deciduous incisors, but no permanent incisors are visible. The dC^1^ have unworn crenulations along their posterior carinae. The dC_1_ is relatively smaller and more incisiform than the C_1_.

In this paper, I explore the postnatal ontogenetic changes in *Smilodon* relative to some extant pantherine felids using geometric morphometric analyses of crania and mandibles, in order to document the nature and magnitude of craniomandibular shape changes during ontogeny in *Smilodon*; the similarities and differences, if any, in ontogenetic shape changes between *S. fatalis* and *S. populator*; and the nature and magnitude of ontogenetic craniomandibular shape changes in the comparative sample of extant pantherines. This will allow a detailed comparison of postnatal ontogenetic craniomandibular shape chances in derived sabertoothed felids relative to extant large felids, and could potentially throw light onto sabercat evolution and to which extant ontogenetic shape changes may mimic evolutionary relationships.

## Materials and Methods

### Data

The database for analysis consisted of the juvenile *Smilodon populator* from Naturhistoriska riksmuseet (NRM) in Stockholm, and 6 crania and 8 mandibles from adult specimens from the Zoological Museum in Copenhagen (CN), the Museum National d'Histoire Naturelle in Paris (MNHN), and the Natural History Museum in London (NHM); three juvenile crania and four juvenile mandibles and 8 adult specimens of *S. fatalis* from the Hancock collection at Los Angeles County Museum (LACMHC), the MNHN in Paris, as well as the author's private collection of casts (PC coll.; a juvenile from Dinocasts, and BC-018T, cast of LACMHC2001-249 from Bone Clones) (Fig. 1,2). The sexes of the included *Smilodon* specimens were unknown, but *Smilodon fatalis* appears to show little, if any, sexual dimorphism [1], [31], which is in contrast to most extant felids, where size-dimorphism is often present, and occasionally also morphological dimorphism [37], [53]–[59].

**Figure 3:**
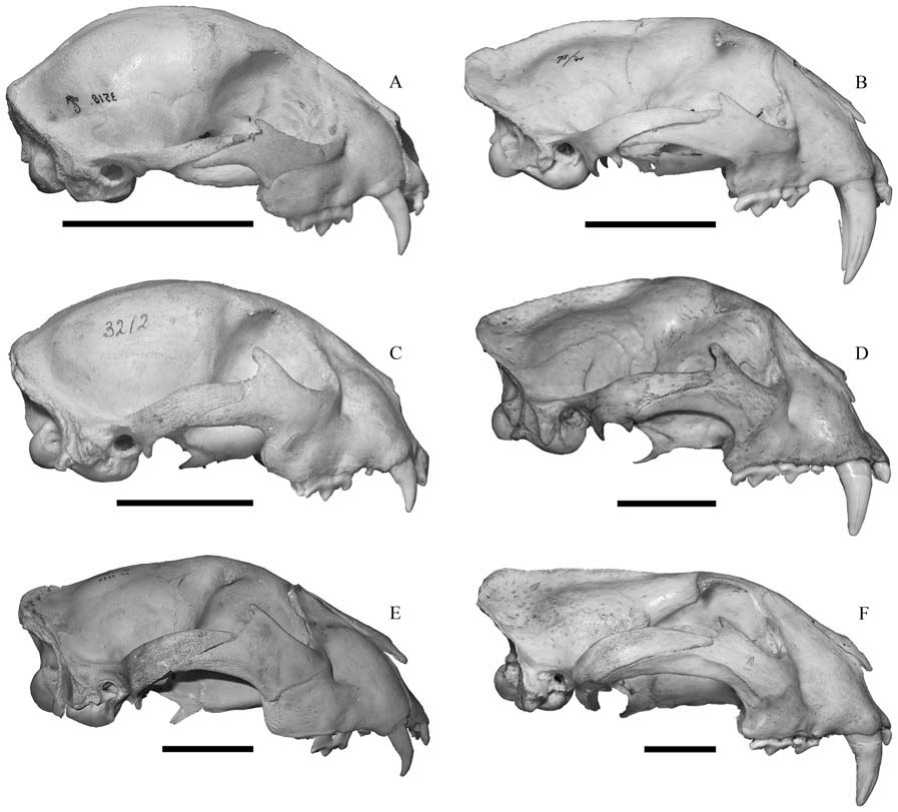
A comparison of crania in juvenile and adult extant felids scaled to the same condylobasal length. A, *Neofelis diardi* juvenile (RMNH3518); B, *Neofelis diardi* adult (RMNH 71/41); C, *Panthera onca* juvenile (RMNH3212); D, *Panthera onca* adult (CN842); E, *Panthera tigris* juvenile (CN5654); F, *Panthera tigris* adult (RMNH “n”). Scale bars equal 5 cm.

**Figure 4:**
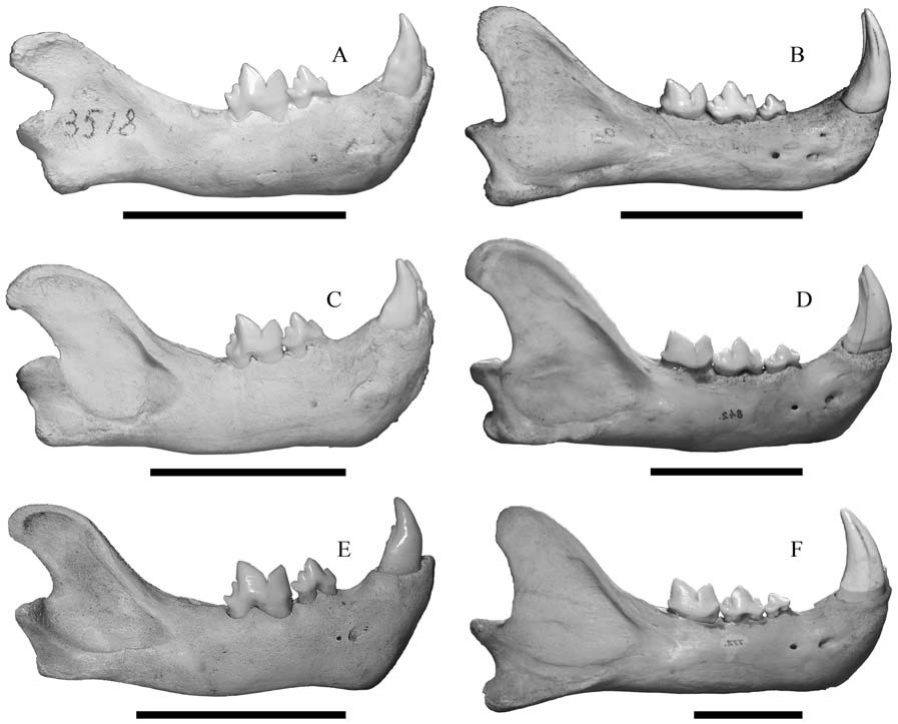
A comparison of mandibles in juvenile and adult extant felids scaled to the same overall length. A, *Neofelis diardi* juvenile (RMNH3518); B, *Neofelis diardi* adult (RMNH “a”); C, *Panthera onca* juvenile (RMNH3212); D, *Panthera onca* adult (CN842); E, *Panthera tigris* juvenile (CN1523); F, *Panthera tigris* adult (CN772). Scale bars equal 5 cm.

As such, the sexes were also mixed in the comparative samples of extant large felids. For comparison were used three pantherines, the Sunda clouded leopard (*Neofelis diardi*; 4 juveniles [1♂; 3♀], and 25 adults [14♂; 11♀]), the jaguar (*Panthera onca*; 4 juveniles [2♂; 2♀], and 50 adults [30♂; 20♀]), and the tiger (*P. tigris*; 4 juveniles [2♂; 2♀], and 50 adults [26♂; 24♀]) (Fig. 3,4). The specimens were from the CN in Copenhagen, NHM in London, MNHN in Paris, the National Museum of Natural History (Naturalis) in Leiden (RMNH), and the author's private collection. More juveniles of *Smilodon fatalis* and the extant large felids were available, but were not included since only juveniles which corresponded in dental ontogenetic stage to the juvenile of *S. populator* were included to ensure comparable ages for analyses of craniomandibular ontogenetic changes.

In *Smilodon fatalis* [48], [50] and *Homotherium serum* [46] the postcanine dental formula in juveniles is dP^3^, dP^4^ (and P^3^, P^4^, and M^1^ in adults) and dP_3_ and dP_4_ (P_4_, M_1_ in adults; *Homotherium* have a reduced P_3_, and this tooth is frequently absent in *Smilodon*). In juveniles, the functional carnassials are dP^3^ and dP_4_, respectively, and the adult carnassials are represented by a small, molariform dP^4^, whereas the dM_1_ does not occur. The deciduous and permanent dentition formula is identical in *Smilodon populator* to *S. fatalis*. The deciduous and permanent dental formulae for the sabercats are also similar to those of extant felids such as leopards [25], lions [27], tigers [37], snow leopards [60], lynxes [29], and other felids [26], except that *Smilodon* and *Homotherium* lack the dP^2^ and P^2^.

In the *Smilodon populator* juvenile and, accordingly, the other juveniles as well, the entire deciduous dentition is fully erupted, and there is no sign of any of the permanent dentition, not even the incisors, which are the first to erupt in sabercats [46], [48], [50] and extant felids [25]–[27], [29], [37], [60]. Tejada-Flores & Shaw [50] divided a growth series of *Smilodon fatalis* from La Brea into five stages for the premaxillae; ten stages for the maxillae; and seven stages for the dentaries. The differences were due to the material often being unassociated. In stage I, all deciduous teeth in premaxilla, maxilla, and mandible were fully erupted, except the dC^1^. Premaxilla and maxilla stages II–IV were not coincident with the same stages in the (un-associated) mandibles, because of the long eruption time of dC^1^. In stage V, all permanent incisors had erupted and the permanent carnassials were in occlusion, and this is too advanced an ontogenetic stage relative to the juveniles used in this analysis. In *S. fatalis*, the dC^1^ is fully erupted when P^4^ is almost erupted, and the C^1^ begins to emerge when the P^4^ is fully erupted and the dP^3^ is about to be shed. This is, as noted, a considerably later ontogenetic stage than the juveniles used in the current analysis. It would appear that the *Smilodon* juveniles in the current analysis correspond to stages II–III.

Age-wise the *Smilodon* juveniles would appear to correspond to Rawn-Schatzingers [46] late stage III (all deciduous incisors and dP^3^ and dP_3_ fully erupted with well developed roots and dP_4_ partly erupted) to early stage IV (all deciduous teeth fully erupted with well-developed roots; wear facets are obvious with concomitant loss of serrations; and deciduous incisors are beginning to be replaced). However, replacement of deciduous incisors has not yet occurred in the juveniles of the current study. Rawn-Schatzinger [46] estimated the age of stage III at 3–4 months and the age of stage IV at 5–12 months, implying that the *Smilodon* juveniles could have been around 4–5 months old. Extant lions with fully erupted deciduous dentition are around 4–5 months old as well, and the first parts of the permanent dentition (incisors) begin erupting at around 7–9 months of age [27], which is similar in tigers [37].

### Geometric morphometrics

To analyze craniomandibular ontogenetic shape changes, digital shape analysis was performed using the Thin Plate Splines (TPS) approach. The TPS function decomposed by its partial warps is a 2D model for analysing shape deformations of structures compared to a predefined reference shape configuration [61]–[63], and may be regarded as a modern model-equivalent of the Cartesian transformation grids for studying evolutionary shape changes originally proposed by Sir D'Arcy Wentworth Thompson (1860–1948) in 1917 [64]. The reference configuration is non-arbitrary and non-local, defining the point of tangency between shape space and approximating tangent space in the computation of the thin plate splines; by default, it is oriented by its principal components axis. It is computed by the generalized orthogonal least squares Procrustes superimposition procedure [62], [65], [66] as a mean reference shape of the included specimens, and, accordingly, it has no morphological, ontogenetic, or phylogenetic significance.

Bending energy is a function of the distance between individual landmarks of the reference configuration and any given specimen being analysed, and it increases progressively with increased localization, i.e. changes affecting only a limited area [62], [63], [67], [68]. The above implies that morphological changes, which may superficially appear to be localised to a particular region and, accordingly, in evolutionary terms uncoupled from the evolution of other structures, are interpreted as localised only if it is required; that is, when adjacent landmarks display contrasting displacements [62], [63], [68], [69]. Accordingly, minimising the amount of spatially localised information leads to more parsimonious interpretation, in that characters are interpreted as evolving independently only when the data require it.

**Figure 5:**
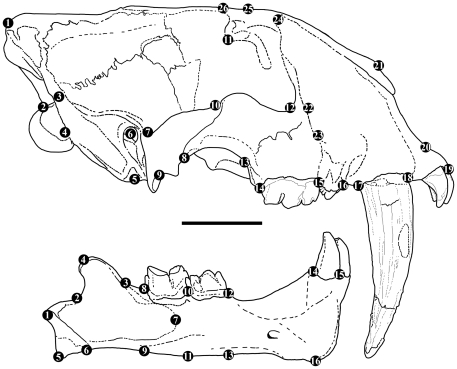
Illustration of a skull of *Smilodon fatalis* (LACMHC2001–3) showing landmarks used in the analysis. Landmarks on the cranium are: 1, top of cranium at the junction of sagittal and nuchal crests; 2, top of occipital condyle; 3, dorsal extent of the mastoid musculature; 4, apex of paroccipital process; 5, apex of mastoid process; 6, centre of external auditory meatus; 7, posterior base of zygomatic arch; 8, ventral junction of jugal-squamosal suture; 9, centre of mandibular condyle; 10, base of postorbital process (jugal portion); 11, apex of postorbital process (frontal portion); 12, centre of orbital aperture; 13, junction of jugal-maxilla suture; 14, posterior, and 15, anterior edge of P^4^ (dP^4^ in juveniles); 15, posterior, and 16, anterior edge of P^3^ (dP^3^ in juveniles); 17, posterior, and 18, anterior edge of C^1^ (dC^1^ in juveniles); 19, anterior edge of premaxilla at incisor alveolus; 20, ventral edge of external narial aperture; 21, apex of nasal; 22, dorsal, and 23, ventral edge of infraorbital foramen; 24, dorsal edge of maxilla-frontal suture; 25, dorsal edge of centre of frontal postorbital process; 26, dorsal edge of beginning of temporal fossa. Landmarks on the mandible are: 1, apex of mandibular cotyle; 2, posterior, and 3, anterior base of coronoid process; 4, apex of coronoid process; 5, posterior, and 6, anterior edge of retroarticular process; 7, anterior extent of mandibular (*M. temporalis*) adductor musculature; 8, posterior, and 10, anterior edge of M_1_ (in the included juveniles, M_1_ is un-erupted but the alveolar orifice and scar can easily be made out); 10, posterior, and 12, anterior edge of P_4_ (dP_4_ in juveniles); 14, posterior, and 15, anterior edge of C_1_ (dC_1_ in juveniles) at the alveolar border; 16, ventral edge of mandibular symphysis; and the depth of the horizontal mandibular ramus posterior to M_1_ (8, 9), at the M_1_/P_4_ junction (10, 11); and anterior to P_4_ (12, 13). Scale bar equals 5 cm.

Twenty-six landmarks were digitized onto each skull and 16 landmarks were digitized onto each mandible in the program tpsDig [70] (Fig. 5). Comparative multivariate analyses on Relative Warp scores, incorporating all included specimens [62], [63], were conducted in tpsRelw [71]. At an α = 0, as used in this study, a Relative Warp analysis is a Principal Component Analysis of shape changes based on the covariance matrix of Partial Warp scores [62], [63], [72]–[74]. The Relative Warps are orthogonal and uncorrelated, and account for virtually all of the variation in the sample.

To analyse net ontogenetic shape changes in juveniles to adults in *Smilodon* and extant pantherine felids multiple specimens within each category (juveniles and adults separately for each taxon) were averaged and ontogenetic shape change analyses from juveniles to adults were conducted in tpsSpline [75]. This approach is often used to study evolutionary shape changes in a selected ingroup of species relative to an outgroup [63], [69]. To facilitate additional comparisons of juvenile to adult shape changes other than Cartesian deformation grids and vector analysis of landmark displacements, the bending energy and the Procrustes Distance, *d*, were computed for each species-pair of juvenile and adult average shape configurations. The bending energy is related to the degree of localisation of landmarks, as noted above, and the Procrustes Distance is an often used metric in pair-wise comparisons of the amount of difference between biological shapes [62], [76].

## Results

**Figure 6:**
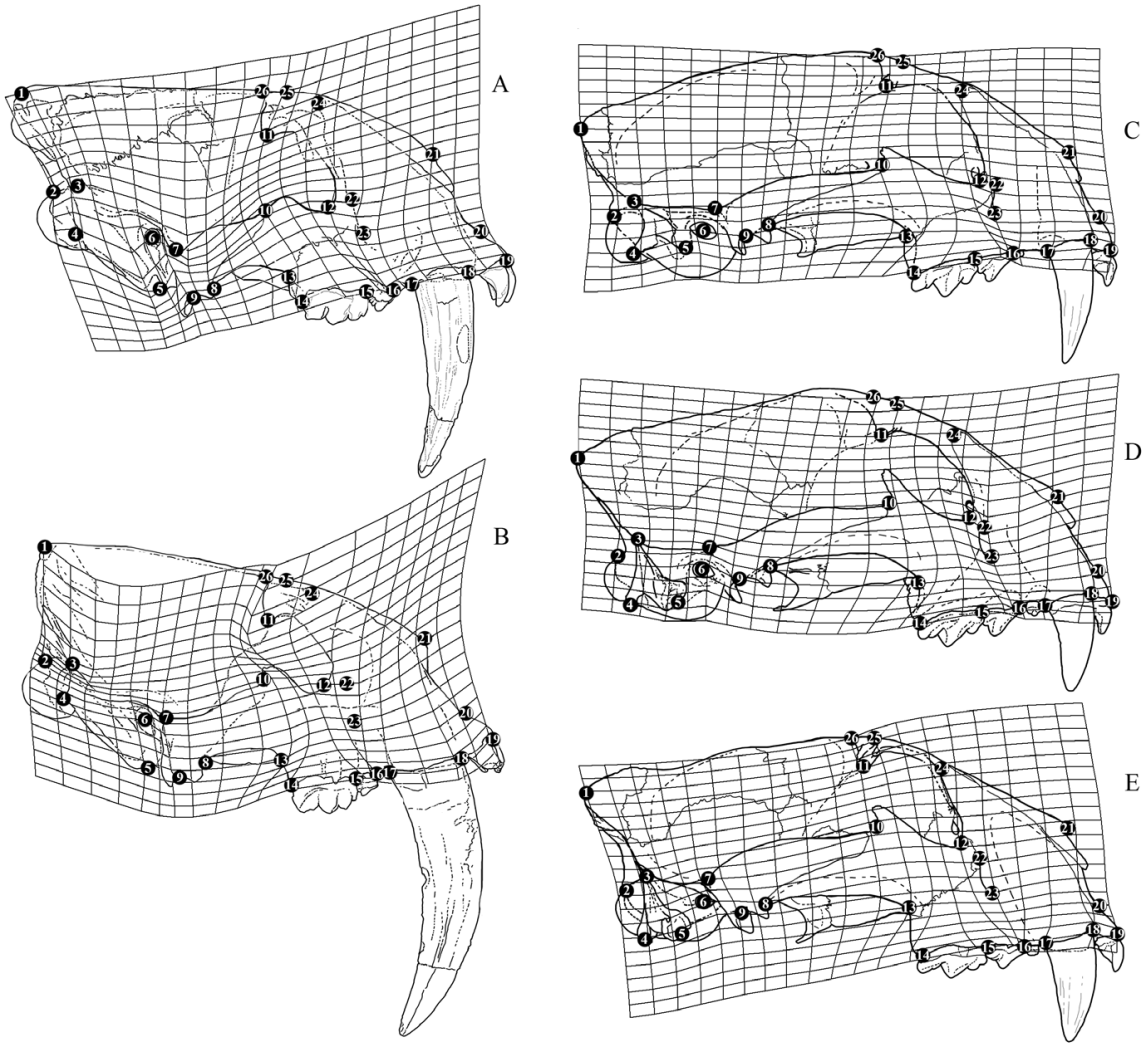
Comparison of cartesian deformation grids illustrating ontogenetic net shape changes in crania of *Smilodon* spp. and extant pantherines. A, *Smilodon fatalis*; B, *S. populator*; C, *Neofelis diardi*; D, *Panthera onca*; and D, *P. tigris*.

Juvenile cranial and mandibular shapes are substantially different from the shapes in adult specimens in each species (Fig. 1, 2, 3, 4) and they occupy different portions of shape space in Relative Warps analysis (Fig. S2,3). Net ontogenetic shape changes in the crania of *Smilodon* are much greater than in extant pantherines, as indicated by vector analysis and the nature of the warp grids of net shape changes (Fig. 6); this is corroborated by much larger Procrustes Distances between juveniles and adults in *Smilodon* compared to extant pantherines. Shape changes are also more localized in *Smilodon* as indicated by much greater bending energies than were computed for extant pantherines. Procrustes Distances of net shape changes between juveniles and adults are 0.1326 in *S. fatalis* and 0.1550 in *S. populator* compared to 0.0687 in *Neofelis diardi*; 0.0833 in *Panthera onca*; and 0.0704 in *P. tigris*. Bending energies are 2.0297 in *S. fatalis* and 3.3846 in *S. populator* compared to 1.4804 in *Neofelis diardi*; 0.9185 in *Panthera onca*; and 1.0249 in *P. tigris*.

Most ontogenetic changes are similar in *Smilodon fatalis* and *S. populator*, but there qre also differences. Both *Smilodon* species show similar overall trends in cranial ontogenetic modularization in that the posterior part of the cranium appears to undergo different shape changes from the anterior part of the cranium (Fig. 6A,B). The facial part of the cranium (landmarks 18–21; 24–26) undergoes a posterodorsal displacement in both species, most notably in *S. populator*, thus causing the palatal region of the skull to become elevated relative to the basicranial region. The mandibular cotyle also becomes more ventrally displaced in the adults (ventral displacement of landmark 9; most strongly in *S. populator*). These two morphological differences from other felids have previously been regarded as key evolutionary adaptations for gaping at high angles to facilitate biting with hypertrophied upper canines [2], [9], [13], [14], and it is here demonstrated that they are ontogenetic changes. It is noteworthy that these shape changes are most strongly expressed in *S. populator*, which on average has larger C^1^ than *S. fatalis*.

In both *Smilodon* species, the already very large mastoid process of the juveniles grows even larger by anteroventral displacement of landmark 5 and posterodorsal displacement of landmark 3, also causing dorsal displacement of the occipital condyle (landmark 2). Consequently, the distance between the anterior tip of the mastoid process and the mandibular cotyle (landmark 9) becomes reduced. In both species, the zygomatic arches expand in anteroposterior length and become more massive in dorsoventral height by anterodorsal displacement of landmark 10 (base of jugal postorbital process) and concomitant anteroventral displacement of landmark 13. In *S. fatalis*, the ventral junction of the jugal-squamosal suture (landmark 8) becomes slightly posteriorly displaced, whereas this is not the case in *S. populator*. There are marked differences in dental proportions from juveniles to adults and replacing the small dP^4^ with the much larger permanent carnassial causes a large posterior displacement of landmark 14 and a large anterior displacement of landmark 15. The width of the C^1^ also increases relative to the dC^1^ by posterior displacement of landmark 17 and anterior displacement of landmark 18. Concomitantly, the P^3^ becomes reduced relative to the functional deciduous carnassial in juveniles.

The dorsal outline of the cranium is straighter and the posterior part of the sagittal crest is more elevated in *S. populator* compared to *S. fatalis* [1], and this is also an ontogenetic difference in that the sagittal-occipital junction (landmark 1) in *S. populator* becomes markedly dorsally displaced whereas it becomes posteriorly and slightly ventrally displaced in *S. fatalis*. In both species, the adults are more prognatheous than the juveniles, as indicated by anterior (*S. fatalis*) and anterodorsal (*S. populator*) displacement of landmark 19. The narial aperture becomes enlarged by dorsal displacement of landmark 21 relative to landmark 20.

In comparison, net ontogenetic cranial shape changes in extant pantherines are substantially less (Fig. 6C–E). In *Neofelis diardi*, the mastoid process grows larger (anterior displacement of landmark 5), and the paroccipital process becomes more posteriorly oriented (posterior displacement of landmark 4); this is different from *Panthera* spp., where the mastoid does not enlarge and the paroccipital faces ventrally, and are traits which *Neofelis* spp. shares with sabercats [13], [14]. Similar changes occur with the zygomatic arches and dentition in extant pantherines as with *Smilodon*, as noted above. Adult extant pantherines also become slightly more prognatheous relative to juveniles (anterior displacement of landmark 19), and although the size of the narial aperture does increase compared to the juveniles, it does so much less than in *Smilodon*. The size of the infraorbital foramen increases slightly in *Panthera* spp., whereas no such changes occur in *Neofelis diardi* or *Smilodon*.

**Figure 7:**
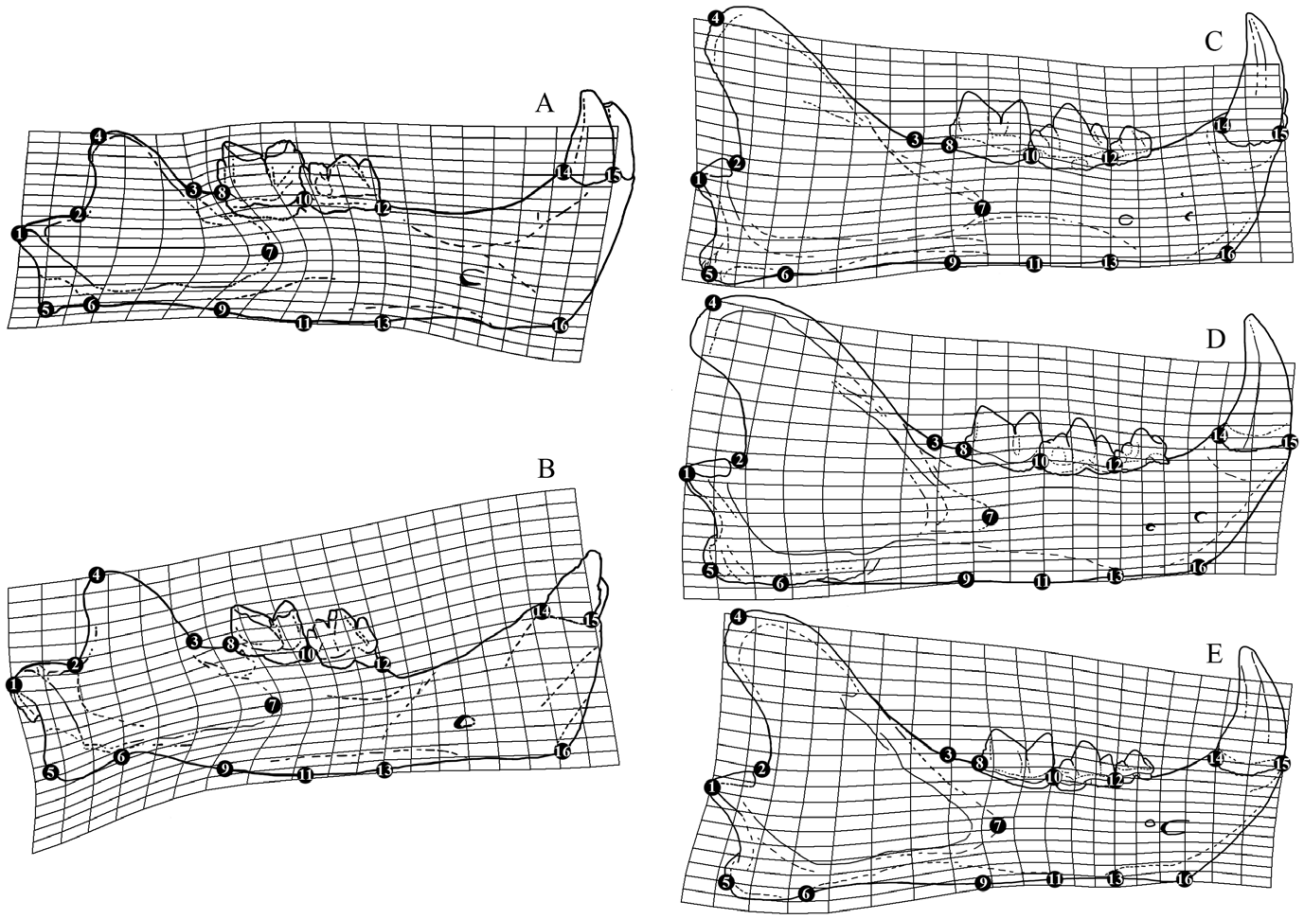
Comparison of cartesian deformation grids illustrating ontogenetic net shape changes in mandibles of *Smilodon* spp. and extant pantherines. A, *Smilodon fatalis*; B, *S. populator*; C, *Neofelis diardi*; D, *Panthera onca*; and D, *P. tigris*.

In contrast, net ontogenetic changes in mandibular shape are notably less in all species (Fig. 7), and the great differences in Procrustes Distance and bending energy between *Smilodon* and the extant pantherines observed in the cranium are not present in the mandible. Procrustes Distances between juveniles and adults are 0.0947 in *S. fatalis* and 0.1021 in *S. populator* compared to 0.0776 in *Neofelis diardi*; 0.1162 in *Panthera onca*; and 0.1289 in *P. tigris*. Bending energies are 0.4029 in *S. fatalis* and 0.1591 in *S. populator* compared to 0.0669 in *Neofelis diardi*; 0.0882 in *Panthera onca*; and 0.1234 in *P. tigris*. The low bending energies imply much less localized shape changes than are present in cranial ontogeny, as confirmed by the warp grids (Fig. 7) and landmark vector analysis.

The mandible also shows modularization in that the posterior part (coronoid process, mandibular condyle, retroarticular process) appears to change independently of the mid-part of the horizontal ramus (dentition portion); and the symphyseal area also undergoes separate ontogenetic shape changes. In *Smilodon*, the symphysis expands dorsoventrally, but not by a large amount, and the mid-part of the horizontal ramus expands slightly dorsoventrally (Fig. 7A,B). Eruption of the permanent carnassial causes anterior displacement of landmark 10 and posterior displacement of landmark 8. The greatest ontogenetic difference is a localized shape change, anterior expansion of the coronoid fossa and attachment for the mandibular adductors, as indicated by a great anterior displacement of landmark 7. The low coronoid of juveniles remains low in adults, and even becomes slightly lower in *S. fatalis* by ventral displacement of landmark 4, whereas no change occurs in *S. populator*. The mandibular condyle becomes further posteriorly offset by a posterior displacement of landmark 1.

Unlike *Smilodon*, where cranial ontogenetic shape changes are much larger than mandibular shape changes, the mandible appears to ontogenetically change equally subtly in extant pantherines as does the cranium (Fig. 7C–E). As in *Smilodon*, the mandible is modularized, and the posterior part undergoes shape changes which appear detached from changes affecting the mid part of the horizontal ramus and the symphyseal region. The most marked shape changes are expansion of the coronoid process by a dorsal (*Neofelis diardi*) or dorsal and slightly anterior (*Panthera* spp.) displacement of landmark 1 and a slight deepening of the posterior coronoid fossa. In contrast to *Smilodon*, the attachment for the mandibular adductors does not increase (i.e., no anterior displacement of landmark 7); the mid-part of the horizontal ramus becomes slightly dorsoventrally constricted rather than expanded, and the symphyseal area does not undergo dorsoventral expansion. In this region is a localized shape change, an expansion of the alveolar width of the C_1_, as evidenced by slight posterior displacement of landmark 14 (all species) and slight anterior displacement of landmark 15 (*Panthera* only). The permanent carnassial causes anterior displacement of landmark 10 and posterior displacement of landmark 8, although less so than in *Smilodon*.

## Discussion

The greatly different cranial morphology of adult *Smilodon* compared to extant felids was brought about by a combination of a juvenile cranial morphology that differed from those of extant pantherine juveniles, as well as postnatal ontogenetic shape changes which exceeded those observed in extant pantherines both in nature and magnitude. In contrast, the greatly different mandibular morphology of adult *Smilodon* compared to extant felids was also present in juveniles and ontogenetic changes were markedly less, and were comparable in magnitude to those observed in extant pantherines. Despite pantherines having a different adult cranial morphology than *Smilodon* and despite their undergoing less radical shape changes during postnatal ontogeny, many of the same areas in the cranium underwent similar postnatal ontogenetic shape changes. Mandibular shape changes were rather subtle and despite differences between *Smilodon* and pantherines, those differences were less than could be surmised from their greatly different adult morphologies.

This would imply that felids, in general, may undergo similar craniomandibular ontogenetic shape changes, and that differences in adult morphology are brought about by a combination of differences in juvenile morphology and the magnitude of ontogenetic shape changes rather than radically different shape changes affecting different craniomandibular areas. This is in agreement with the findings of Goswami [77] who demonstrated similar patterns of cranial landmark integrations among felids, including *Smilodon*. Some areas, however, underwent changes in *Smilodon* but not *Panthera*, such as mastoid size, and it is noteworthy that the Sunda clouded leopard, a taxon which has a number of craniomandibular and dental features in common with basal sabercats [13], [14], also underwent ontogenetic changes in this area, albeit less markedly. The above suggests that the ontogenetic pattern established for *Smilodon* could be characteristic of all derived sabercats and this may be tested by analyses of the few species where juveniles are known, notably the homotherines *Amphimachairodus giganteus* and *Homotherium serum*.

*Smilodon* and extant pantherines show modularization of postnatal ontogenetic shape changes along the same lines that have previously been documented for other mammals. Modularization of the alveolar (including dentition) portion of the mandible from the rest of the mandibular corpus appears to be an ancient and basic mammalian pattern [78], [79], and has been documented in, for instance, mice [80], [81], and a variety of primates, such as lorises [82], cercopithecines [83], great apes [84], and humans [85]. In general, the structures associated with the mandibular adductors (coronoid process; posterior part of horizontal ramus) appear to form a rather well integrated unit in a variety of mammals. As such, it appears reasonable to extend those inferences to felids as well, such as *Smilodon* and other sabercats, as well as extant felids, which is corroborated by the findings of this study. However, in primates, the ontogenetic mandibular integration of modules is not necessarily accompanied by similarities in cranial integration, and different parts of the cranium even in closely related species may have different patterns of cranial character integration, in particular in the region of the facial skeleton and degree of prognatism [85]–[90]. This indicates that among great apes (including humans), the mandible has undergone less evolutionary ontogenetic changes than the cranium [85]. This is also the case in *Smilodon* but not extant pantherines.

It has long been realized that the widespread notion in late 19^th^–early 20^th^ century biological sciences that ontogeny recapitulates phylogeny is, at best, oversimplified and in many instances simply incorrect [91]–[94] in the sense presented by Ernst Haeckel (1834–1919) in his dually famous and infamous Biogenetic Law, first presented in full in his 1868 contribution, *Natürliche Schöpfungsgeschichte* [95]. However, it has been demonstrated that vertebrate embyos do bear a strong, if in some instances superficial resemblance to one another in early ontogeny, increasing their morphological differentiation through later ontogny, and post-natal juveniles in many groups of closely related mammals often bear a closer resemblance to each other structurally than do the adults [92], [93], [96]. In several mammalian groups, characters that appear early in phylogeny also often appear at an earlier stage during ontogeny [92], [93]; a famous example is the fossiliferously well documented equid evolution and the correlates with studies of postnatal ontogny [97]–[99].

Specialized, Plio-Pleistocene sabertoothed felids are substantially different from basal Miocene species in terms of craniomandibular and to a lesser extent dental morphology [2], [12]–[17], [19], [20], [100], [101]. The *Smilodon* juveniles do not in most respects bear much resemblance to adults of basal sabercats such as *Machairodus*, *Promegantereon* [102], *Paramachairodus*, *Dinofelis*, or *Metailurus*, and, as such, their ontogeny does not reflect phylogenetic shape changes among adult sabercats, but their overall, more elongate cranial shape is clearly plesiomorphic in that this was also the case among adults of basal sabercats but not adult *Smilodon*. In other respects, *Smilodon* juveniles are clearly morphologically derived, as the adults, for instance in having a very low coronoid process, a very large mastoid process, and large, blade-like upper canines and very short, incisiform lower canines. However, it is clear that in some respects, postnatal ontogeny in *Smilodon* does resemble, if not exactly recapitulate, phylogeny (see phylogeny in [8]).

The palatal part of the skull is far more upturned in derived sabercats than in basal sabercats, and this is an ontogenetic change in *Smilodon* accomplished by posterodorsal displacement of anterior landmarks 18–21 and 24–26 relative to the basicranial landmarks. The mandibular cotyle is far more ventrally displaced in *Smilodon* adults than in basal sabercats, and this also takes place during postnatal ontogeny by ventral displacement of landmark 9. Both of these morphological differences from extant felids have previously been regarded as key evolutionary adaptations for gaping at high angles to facilitate biting with hypertrophied upper canines [2], [9], [13], but it has hitherto been unknown if they constituted ontogenetic changes. Basal sabercats are less prognatheous than derived sabercats, most notably the homotherines (e.g., *Homotherium*, *Xenosmilus*), but also *Smilodon*, and landmark 19 becomes anteriorly (*S. fatalis*) or anterodorsally (*S. populator*) displaced during postnatal ontogeny in *Smilodon*. Enlargement of the narial aperture also occurs ontogenetically, and in basal sabercats the narial aperture is relatively smaller than in derived species, including *Smilodon*. The mandibular condyle (mandible landmark 1) is more posteriorly displaced in derived sabercats, and is another instance of *Smilodon* ontogeny recapitulating phylogeny; this trait is believed to be another adaptation for achieving high gape angles [9], [20].

Efficient biting and high bite forces are key adaptations for predation among carnivores, and unsurprisingly, in the puma, the most prevalent craniomandibular ontogenetic changes are those affecting portions of the cranium and mandible which are directly associated with the more demanding feeding ecology experienced when gradually substituting suckling for large vertebrate predation. Accordingly, during ontogeny puma skulls become taller with well developed occipital and sagittal crests, and relative postorbital width decreases, thus creating more space for the *m. temporalis*; zygomatic width increases; the muzzle become more sturdy; and the coronoid process increases [39], [40]. The findings of these authors for the puma are corroborated by the results for the pantherines of the current study, and, as such, the suggestion of Peigné & Bonis [103] that juveniles of leopard, serval, and caracal have more backwards-oriented coronoid processes than adults, but that coronoid process height does not change ontogenetically is not corroborated by the present study or by the findings of Giannini et al. [40]. This similarity of ontogenetic patterns among felids is to be expected, since felids are strictly carnivorous, and, accordingly, Goswami [77] found no correlation of cranial landmark integration in felids, including *Smilodon*, whereas this was present in Caniformia, Arctoidea and Musteloidea (including Mustelidae), which have a wider range of diets and, thus, presumably have undergone more differentiated evolutionary adaptations to accompany their dietary diversity.

Postnatal ontogenetic shape changes in *Smilodon* appear also to have increased biting efficiency and power, for instance in raising the palatal region to facilitate greater clearance between the upper and lower canines. The skull becomes taller and the zygomatic arches expand in anteroposterior direction (more space for the *m. temporalis*), but the arches also become dorsoventrally taller, thus creating a larger insertion area for the *m. masseter*, which would provide the arches with greater mechanical resistance to the action of the masseters. The attachment for the mandibular adductors on the mandibular horizontal ramus expands anteriorly; the coronoid process becomes anteroposteriorly expanded; the mandibular horizontal ramus becomes more sturdy, presumably so as to become more resistant to forces from biting [22]; the already large mastoid process in juveniles enlarges even further; and the occipital condyle is lifted, thus providing more leverage for the important head depressing action during predation [2], [10], [12], [20]. These ontogenetic shape changes would appear all to be tied to the mechanically more demanding actions of vertebrate predation than suckling, as they imply increased biting power. The upper canine also grows much longer but also more anteroposteriorly sturdy. However, in contrast to extant felids, the coronoid process does not get taller, presumably since a low coronoid process was an important adaptation for achieving very high gape angles [2], [9], [10], [12]–[16], [18], [20].

Morphological variation in the voluminous La Brea material of *Smilodon fatalis* adults is substantial [31], [47], [104], and this was part of the reasons for Berta [6] synonymizing the Pleistocene North and South American *Smilodon* into one species, *S. populator*. Kurtén & Werdelin [1] provided a comprehensive analysis showing that consistent differences were present, and that this indicated two species, *S. fatalis* from North and north-western (Pacific coast) South America, whereas *S. populator* occupied the remainder of the South American continent. To their species differences may be added several ontogenetic differences, such as greater palatal elevation and greater mandibular cotyle depression in *S. populator*; and posterior displacement of the ventral junction of the jugal-squamosal suture in *S. fatalis*. The straight dorsal cranial profile in *S. populator* was a character upon which Kurtén & Werdelin [1] placed emphasis as species-distinction from *S. fatalis*, and the current study expands on this finding, demonstrating that it is a postnatal ontogenetic shape difference between the two species. Accordingly, craniomandibular ontogeny also indicates differences between the eastern South American and the North American Pleistocene material of *Smilodon*, corroborative of two species.

## Supporting Information

Figure S1 **Juvenile specimen of** ***Smilodon populator*** **from Naturhistoriska riksmuseet in Stockholm.**
(DOC)

Figure S2 **Relative Warps analysis of juvenile and adult cranial shape in** ***Smilodon*** **spp. and extant pantherines.**
(DOC)

Figure S3 **Relative Warps analysis of juvenile and adult mandible shape in** ***Smilodon*** **spp. and extant pantherines.**
(DOC)
