# Supplementary material for: The Making of a Monster: Postnatal Ontogenetic Changes in Craniomandibular Shape in the Great Sabercat Smilodon
Source: PLoS One. 2012 Jan 3;7(1):e29699. doi: 10.1371/journal.pone.0029699 (PMC3250457; doi:10.1371/journal.pone.0029699)

**Figure S1**

Juvenile specimen of *Smilodon populator* from Naturhistoriska riksmuseet in Stockholm in lateral (A, left; B, right) view; C, ventral view; D, anterior view; E, posterior view; F, lateral view of mandible; G, anterior view of mandible. Condylobasal length of this specimen is 198.5 mm, compared to a range of adults of 290-385 mm (n=6). Scale bar equals 5 cm.


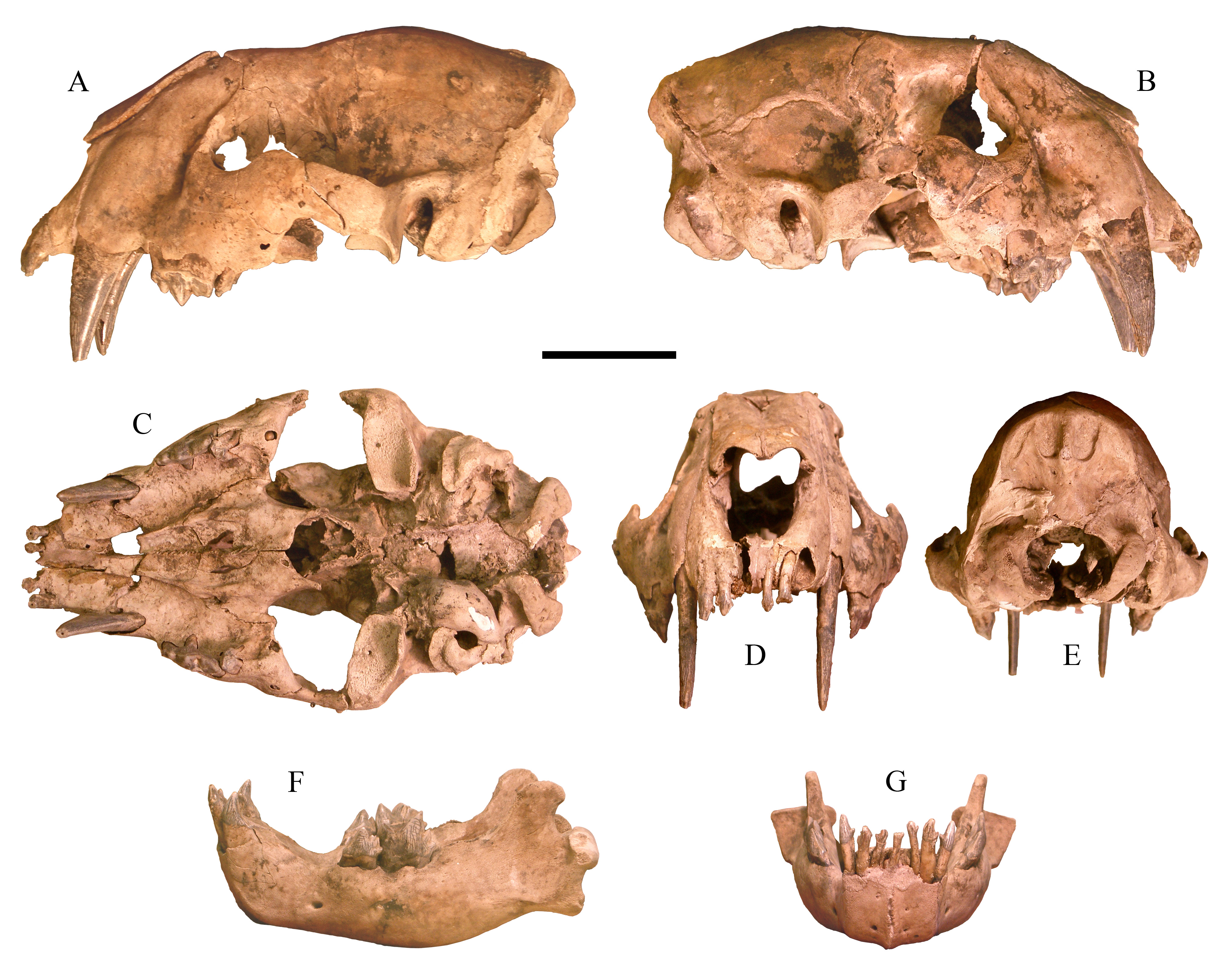

Supplement: Figure S1 — Juvenile specimen of Smilodon populator from Naturhistoriska riksmuseet in Stockholm. (DOC) [file pone.0029699.s001.doc]
