# Supplementary material for: The Making of a Monster: Postnatal Ontogenetic Changes in Craniomandibular Shape in the Great Sabercat Smilodon
Source: PLoS One. 2012 Jan 3;7(1):e29699. doi: 10.1371/journal.pone.0029699 (PMC3250457; doi:10.1371/journal.pone.0029699)

**Figure S3**

Relative Warps analysis of juvenile and adult mandible shape in *Smilodon fatalis*, *S. populator*, *Neofelis diardi*, *Panthera onca*, and *P. tigris*. Open symbols denote juveniles and filled symbols denote adults.


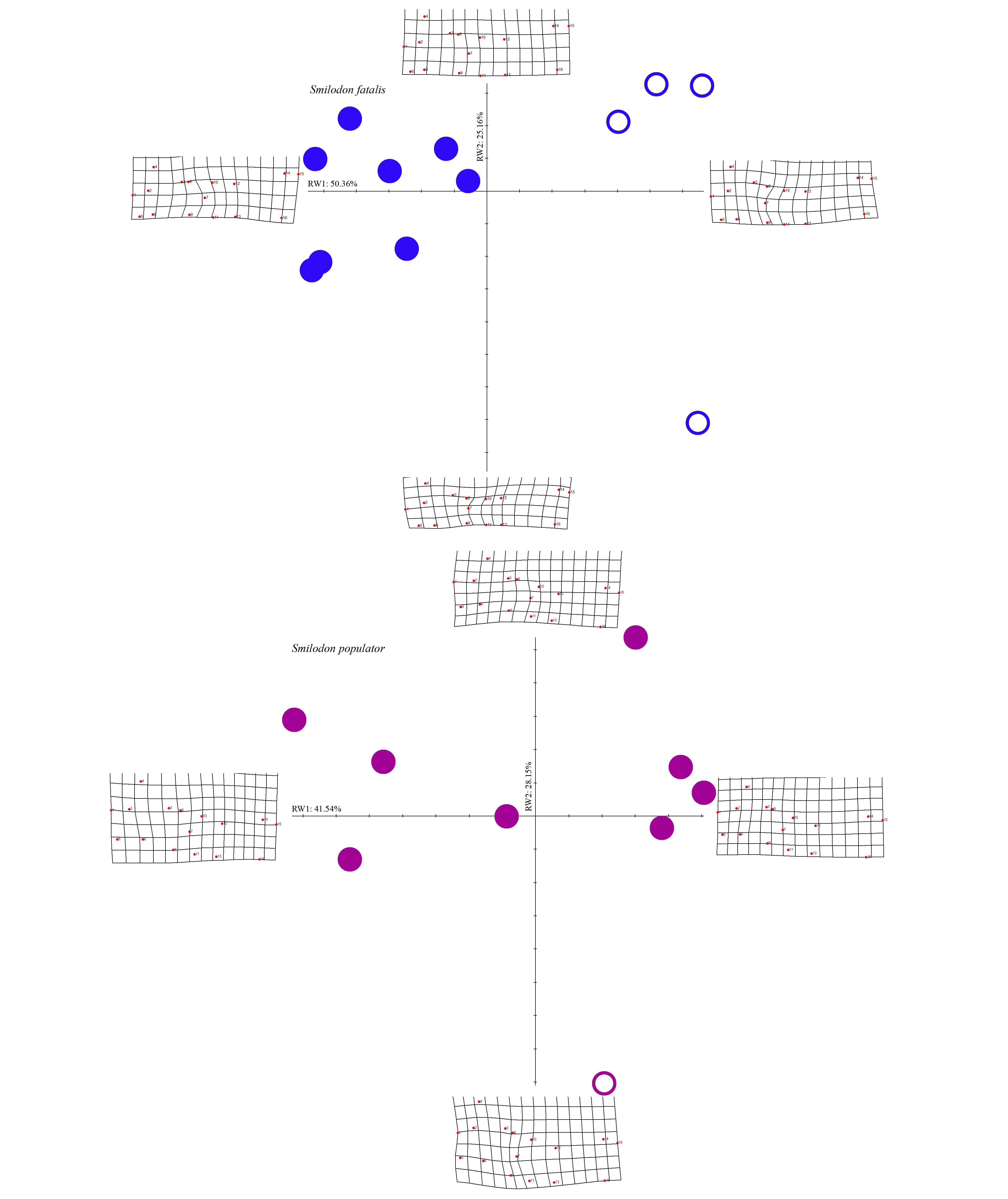


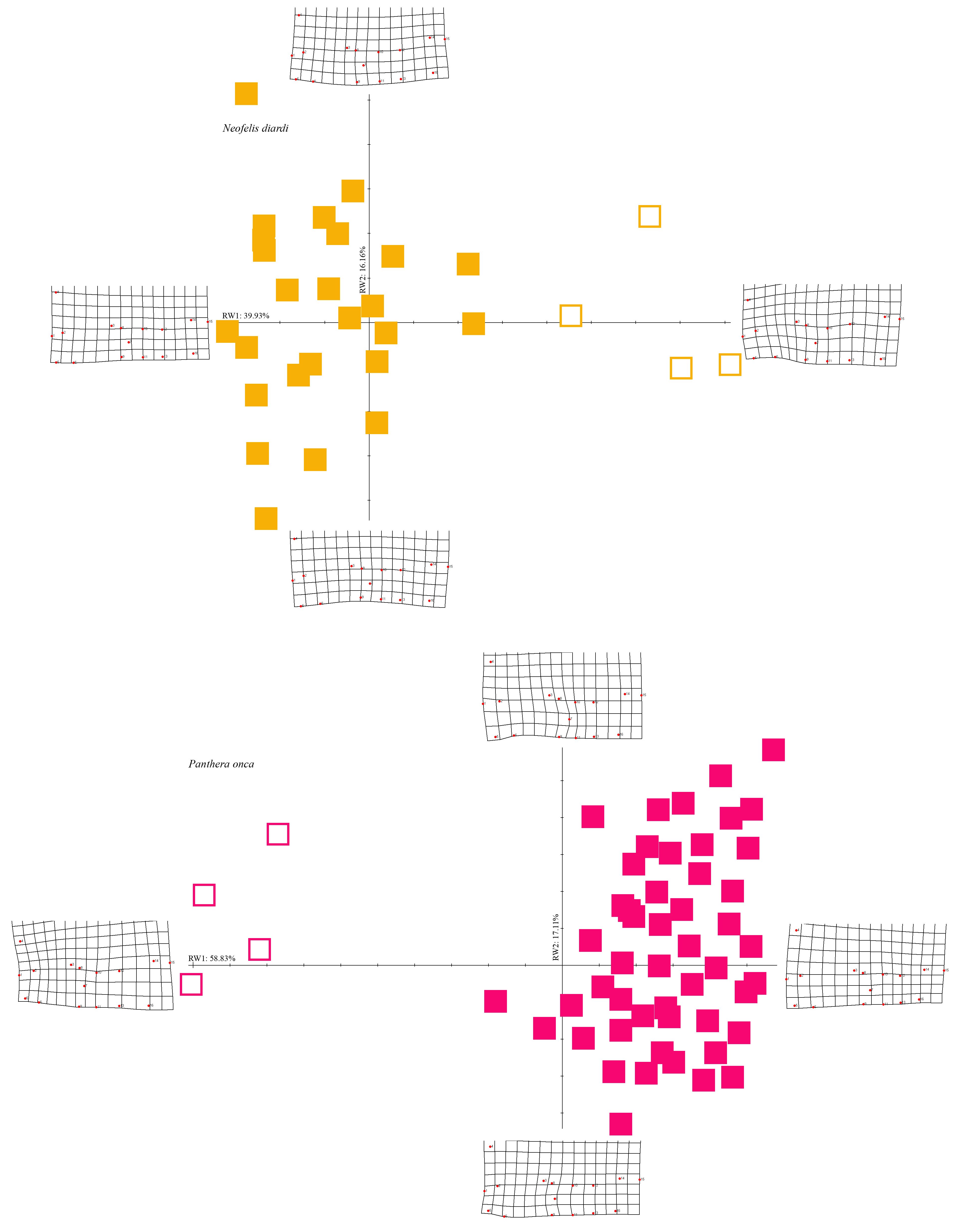


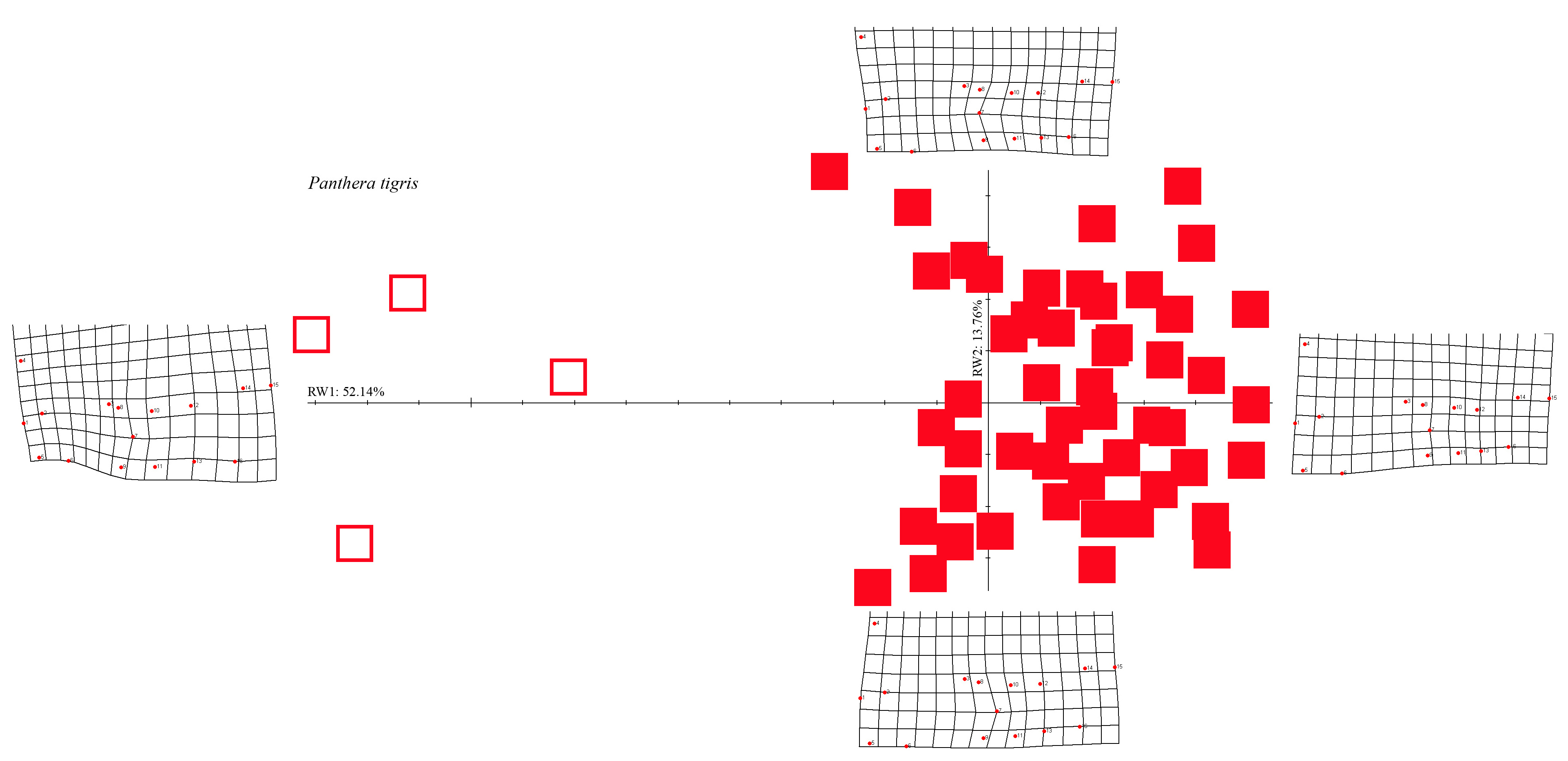

Supplement: Figure S3 — Relative Warps analysis of juvenile and adult mandible shape in Smilodon spp. and extant pantherines. (DOC) [file pone.0029699.s003.doc]
